# Role of reaction kinetics and mass transport in glucose sensing with nanopillar array electrodes

**DOI:** 10.1186/1754-1611-1-5

**Published:** 2007-10-10

**Authors:** Venkataramani Anandan, Xiaoling Yang, Euihyeon Kim, Yeswanth L Rao, Guigen Zhang

**Affiliations:** 1 Micro/Nano Bioengineering Lab, Department of Biological and Agricultural Engineering, The University of Georgia, Athens, GA 30602, USA; 2 Department of Biomedical Engineering, Georgia Tech and Emory University, Atlanta, GA 30332-0535, USA; 3 Nanoscale Science and Engineering Center, The University of Georgia, Athens, GA 30602, USA; 4 Faculty of Engineering, The University of Georgia, Athens, GA 30602, USA

## Abstract

The use of nanopillar array electrodes (NAEs) for biosensor applications was explored using a combined experimental and simulation approach to characterize the role of reaction kinetics and mass transport in glucose detection with NAEs. Thin gold electrodes with arrays of vertically standing gold nanopillars were fabricated and their amperometric current responses were measured under bare and functionalized conditions. Results show that the sensing performances of both the bare and functionalized NAEs were affected not only by the presence and variation of the nanoscale structures on the electrodes but also by the reaction kinetics and mass transport of the analyte species involved. These results will shed new light for enhancing the performance of nanostructure based biosensors.

## Background

Biosensors are important devices for monitoring biological species in various processes of environmental, fermentation, food and medical concerns. The main challenges biosensors face include low sensitivity, poor specificity and proneness to fouling. The advent of nanotechnology presents some promising solutions for alleviating these problems. While many efforts have been devoted to improving the performances of biosensors by taking advantage of nanostructures or macrostructures with nanoscale features, the effort to elucidate the underlying mechanism governing the performances of biosensors enhanced with nanostructures is still scant.

This study intends to investigate the role of reaction kinetics and mass transport in biosensing when electrodes with nanoscale features are used. For this purpose, we used glucose biosensor as a model system. In a typical glucose biosensor, an enzyme such as glucose oxidase is immobilized onto the electrode surface [1,2]. The performance of such functionalized electrodes can be improved by either adjusting the spatial distribution of the enzyme or by modifying the morphology of the electrode surface. To achieve a high efficiency in immobilizing an enzyme onto the electrode surface, various techniques have been developed, such as the use of self-assembled monolayer [1-4], conducting polymers [5,6] and sol-gels [7]. Among these methods, the self-assembled monolayer (SAM) approach offers a better control for enzyme distribution at the molecular level, a high degree of reproducibility in enzyme immobilization and a short distance between the immobilized enzyme and the electrode surface [1,4]. The SAM approach, however, is limited by the amount of the enzyme that can be immobilized onto the electrode surface, which in turn will affect the sensing performance of the biosensor [8]. To increase the amount of immobilized enzyme various nanostructures such as nanotubes, nanoparticles and nanorods have been explored in order to increase the active surface area of the electrodes. For example, nanostructures like gold nanotubes [8], carbon nanotubes [5,9] and gold nanoparticles [10] have been incorporated into electrode surfaces and they exhibited better performance than conventional flat electrodes.

Recently Wang et al. [11] used nanostructured platinum electrodes functionalized with glucose oxidase for glucose detection. These electrodes showed a significant (two orders of magnitude) increase in glucose detection sensitivity as compared with a flat electrode, but the response of these electrodes to K_4_Fe(CN)_6 _was just 2.3 times that of the flat electrode. They attributed such sensitivity enhancements for glucose detection to the increased enzyme loading and improved retention of hydrogen peroxide near the electrode surface without examining systematically the role of reaction kinetics and mass transport. We believe that the electrical current response of these nanostructured electrodes is controlled by reaction kinetics, mass transport and the geometric topography of the nanostructures. Thus, to be able to understand the mechanism governing such an electrochemical process for the purpose of improving the performance of nanostructure based electrodes, it is necessary to investigate the role of reaction kinetics and mass transport in biosensing when nanostructured electrodes are used.

### Experimental Methods

Nanopillar array electrodes (NAEs) with three different pillar heights were fabricated using a template method [12]. In fabricating these electrodes, a layer of gold film about 150 nm thick was first sputter-coated onto one side of a porous anodic alumina (PAA) circular disc (d = 25 mm; Whatman Inc, Maidstone, England) having an average pore diameter of 150 nm using a SPI sputter coater (Structure probe Inc, West Chester, PA). Then, a thicker gold layer was electrodeposited on top of the sputtered gold film to form a strong supporting base in an Orotemp24 gold plating solution (Technic Inc, Cranston, Rhode Island) with a current density of 5 mA/cm^2 ^for two minutes. This supporting base was masked with Miccrostop solution (Pyramid plastics Inc., Hope, Arkansas) for insulation. After that, gold nanopillars were electrodeposited through the open pores of the PAA disc from the uncoated side under an electrical current density of 5 mA/cm^2 ^at 65°C. The deposition time was varied for achieving nanopillars of different heights. For this study, specimens with three different nanopillar heights were prepared with the electrodeposition time controlled at 1, 7 and 15 minutes. After nanopillar deposition, the PAA disc was dissolved in 2.0 M NaOH resulting in a thin gold sheet with arrays of vertically standing gold nanopillars. The fabricated specimens were cut into small square pieces (about 3.2 × 3.2 mm^2^) and they were grouped into specimens A, B and C by their nanopillar height. For connecting the electrodes, a copper tape were attached to the backside of an electrode with the exposed part of the copper tape insulated using Miccrostop. Of these small specimens, some were used for scanning electron microscopy (SEM) imaging analyses, and some for electrochemical experiments (bare and functionalized conditions). For controls, two flat gold electrodes (one for bare and one for functionalized condition) with the same geometric size were prepared by depositing a thin film (300 nm) of gold on a titanium coated glass plate using a thermal evaporator (built in house). Prior to the electrochemical experiments, all electrodes (NAEs and flat) were cleaned by running cyclic voltammetry (CV) in 0.3 M H_2_SO_4 _between -500 mV and 1500 mV until a stable CV curve was obtained for each specimen, and then washed with deionized water.

These electrodes were characterized in either bare or functionalized conditions. In the bare condition the cleaned electrodes were used directly, and in the functionalized condition the cleaned electrodes were further functionalized prior to use. To functionalize the electrodes, their surfaces were first modified with a SAM layer by placing them in a 75% ethanol solution containing 10 mM 3-mercaptopropionic acid. Then the SAM modified electrodes were rinsed in 75% ethanol and immersed in a 0.1 M 2-(N-morpholino) ethanesulfonic buffer solution (pH of 3.5) containing 2 mM 1-ethyl-3-(3-dimethylaminopropyl) carbodiimide hydrochloride and 5 mM N-hydroxysuccinimide for activation for two hours. After washing in phosphate buffer solution (PBS), the activated NAEs were placed in PBS solution at pH 7.4 containing 1 mg/ml of glucose oxidase for two hours under constant stirring. The reason for setting the immobilization time to two hours is that according to literature [13], enzyme loading reaches its maximum in about 2 hours and it saturates afterwards. From the electrochemical experiments, the amperometric current responses of both bare and functionalized NAEs along with flat controls were measured using a conventional three-electrode cell with an Ag/AgCl reference electrode and a platinum counter electrode with the Multistat 1480 (Solartron Analytical, Houston TX, USA) electrochemical system.

For the bare-electrodes, their amperometric current responses at different concentrations of K_4_Fe(CN)_6 _to each incremental addition of 80 μl of 1 M K_4_Fe(CN)_6 _to a 20 ml solution containing 0.5 M Na_2_SO_4 _(equivalent to a 4 mM increase in K_4_Fe(CN)_6_concentration) were measured at a constant electrode potential of 350 mV (vs. Ag/AgCl), and the change in the current response upon the change in K_4_Fe(CN)_6 _concentration for both the NEAs and flat electrode was determined. For the functionalized NAEs, the amperometric current responses to each incremental addition of 50 μl of 1 M glucose to a 20 ml PBS solution (equivalent to a 2.5 mM increase in glucose concentration) containing 3 mM p-benzoquinone as a mediator were measured at a constant potential of 350 mV (vs. Ag/AgCl). In all experiments, the background current of all electrodes was allowed to stabilize before drops of target species were added. Prior to these experiments the electrolyte solution was deaerated with nitrogen and during experiments the solution was blanketed with nitrogen and stirred constantly at 600 rpm.

For glucose detection, the electrode reactions in the present study can be described by the following cascading events [1]. With the catalysis of glucose oxidase (GOX-FAD), glucose was first oxidized into gluconolactone with GOX-FADH_2 _as a by-product. The GOX-FADH_2 _was then converted back to its oxidized form (GOX-FAD) by the p-benzoquinone mediator in the solution. The mediator itself was converted back to its original form by oxidation at the electrode surface, through which free electrons were generated and picked up by the electrode to produce a current response. These cascading events can be expressed by the following reactions:

Glucose + GOX-FAD → Gluconolac tone + GOX-FADH_2_

GOX - FADH_2 _+ 2 Mediator_ox _→ GOX - FAD + 2 Mediator_red _+ 2H^+^

2Mediator_red _→ 2Mediator_ox _+ 2e^-^

Based on these enzymatic events and the current measurements from the functionalized electrodes, we performed an enzymatic-kinetics study to determine the enzyme activity in the functionalized electrodes by using the Michaelis-Menten equation:

Is=Imax⁡SKm+S
 MathType@MTEF@5@5@+=feaafiart1ev1aaatCvAUfKttLearuWrP9MDH5MBPbIqV92AaeXatLxBI9gBaebbnrfifHhDYfgasaacH8akY=wiFfYdH8Gipec8Eeeu0xXdbba9frFj0=OqFfea0dXdd9vqai=hGuQ8kuc9pgc9s8qqaq=dirpe0xb9q8qiLsFr0=vr0=vr0dc8meaabaqaciaacaGaaeqabaqabeGadaaakeaacqWGjbqsdaWgaaWcbaGaem4Camhabeaakiabg2da9maalaaabaGaemysaK0aaSbaaSqaaiGbc2gaTjabcggaHjabcIha4bqabaGccqWGtbWuaeaacqWGlbWsdaWgaaWcbaGaemyBa0gabeaakiabgUcaRiabdofatbaaaaa@3BF1@

Where *I*_*s *_is the steady-state current measured at each glucose concentration, *I*_max _the maximum current attainable, *K*_*m *_the apparent Michaelis-Menten constant, and *S *the concentration of the target species (i.e., glucose in this case). The parameter *K*_*m *_describes the enzymatic activity of glucose: the smaller the *K*_*m *_value is, the more efficient the enzymatic reaction is. When *K*_*m *_of an enzymatic biosensor is larger than the *K*_*m *_value of the freely dissolved enzyme, it usually implies that the enzyme immobilized in the biosensor is less efficient in oxidizing glucose than the dissolved enzyme [14]. In this study, the *K*_*m *_values for the functionalized electrodes were determined by performing nonlinear curve fit using Eq.4 to the measured current-concentration data.

### Simulation Procedures

**Figure 1:**
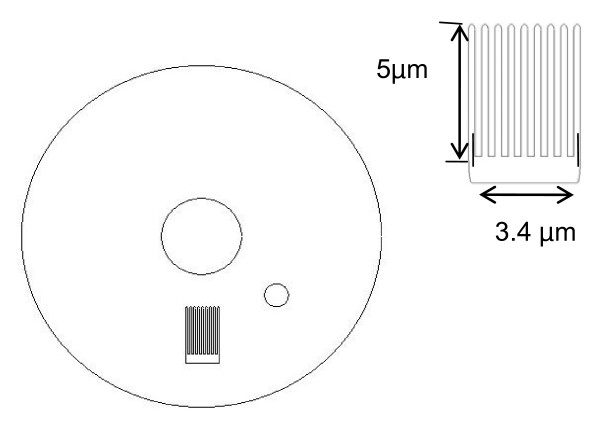
A 2D model of a circular electrochemical cell containing a functionalized nanopillar electrode. The inner center circle is for generating the swirling vortex force to stir the solution and the small off-center circle is for creating a drop of uniform concentration of glucose prior to the kinetics analysis. A magnified view of the nanopillar electrode is shown at the upper right corner.

To elucidate the effects of reaction kinetics and mass transport on the current response of bare and functionalized NAEs, we simulated such an electrochemical process using a finite element analysis method with commercial software COMSOL Multiphysics (COMSOL Multiphysics, Burlington, MA). To simplify the situation we considered two dimensional situations. As shown schematically in Figure 1, a set of NAEs (with a width and a spacing of 200 nm for the pillars, and an overall dimension of 5 μm × 4.3 μm for the electrode) was placed in a circular electrochemical cell containing a supporting electrolyte. In this simulation, a bare and a functionalized (with glucose oxidase) NAEs as well as a flat electrode with the same planar area (as a control) were considered.

For the electrode reaction at the functionalized NAEs, we assumed that glucose was consumed at a flux of *J*_*g *_at the electrode surface to produce the mediator in its reduced-form at a flux of *J*_*M*_. Here *J*_*g *_and *J*_*M *_can be described by the following equations:

*J*_*glucose *_= *kc*_*G *_

*J*_*M *_= *kc*_*G *_- *k*_0_*c*_*M *_exp(-*αF*(*E *- *E*_*std*_)/*RT*)

where *k *represents the rate constant for Eq. 1, *c*_*G *_the concentration of glucose, *c*_*M *_the concentration of mediator, *k*_0 _the standard rate constant, *α *the charge transfer coefficient, *F *the Faraday constant, *E *the electrode potential, and *E*_*std *_the standard potential of the mediator. To simulate the actual event, the electrode was held at a constant overpotential of 350 mV. Under this condition, the reduced-form mediator was oxidized at the electrode surface to generate a current flux of *J*_*c*_:

*J*_*C *_= -2*k*_0_*c*_*M *_exp(-*αF*(*E *- *E*_*std*_)/*RT*)

With these considerations, the amperometric current response of the electrodes in response to a drop of glucose was determined while the electrolyte solution was constantly stirred by a swirling vortex force applied at the center of the cell.

For the electrode reaction at the bare-electrode, we considered the redox of K_4_Fe(CN)_6 _with the reduction flux of K_4_Fe(CN)_6 _governed by:

*J*_*F *_= -*k*_0*F*_*c*_*F*1 _exp(-*αF*(*E *- *E*_*std*_')/*RT*) + *k*_0*F*_*c*_*F*2 _exp(-*αF*(*E *- *E*_*std*_')/*RT*)

where *k*_0*F *_is the electron transfer rate for both ferrocyanide and ferricyanide (assumed to be the same), *c*_*F*1 _the concentration of ferrocyanide, *c*_*F*2 _the concentration of ferricyanide, *E *the electrode potential, and *E*_*std*_' the standard potential of ferro- and ferri-cyanide.

Besides the reaction kinetics discussed above, the mass transport in these electrochemical processes was mainly governed by diffusion and convection for the mobile species such as glucose and K_4_Fe(CN)_6_. The electromigration was ignored because of the presence of the supporting electrolyte in a high concentration.

After these considerations, the diffusion/convection-controlled electrochemical reaction problems upon a step potential excitation (350 mV) at the electrode were solved using the combined Electrokinetic-Flow and Navier-Stokes applications in COMSOL Multiphysics. In the simulation process, two initial analyses were performed. First, a stationary nonlinear analysis in Navier-Stokes mode was performed for reaching a fully developed vortex flow inside the center inner circle (see Figure 1), and then a stationary nonlinear analysis in Electrokinetic-Flow mode was performed for producing a uniform initial concentration of glucose within the off-center inner circle (see Figure 1), much like dropping a small volume of glucose into the solution. After these initial steps, time dependent analyses were performed. For the kinetic constants, literature values [15] including the diffusivity of ferrocyanide and ferricyanide listed in Table 1 were used. The values for the diffusivity of glucose and the mediator, which are not readily available in the literature, were calculated using the following equation [16]:

**Table 1:** Material constants and kinetic parameters used in the simulation [15]

Parameter		Value
*k*_0_	Standard rate constant	1.5 × 10^-3 ^(m/s)
*ε*_*B*_	Association factor	2.6
*α*	Charge transfer coefficient	0.5
T	Absolute temperature	298 (K)
*μ*	Viscosity	1.1 (cP)
*V*_*A*_	Molar volume	0.1176 (m^3^/mol)
*r*_*p*_	Pore radius	200 × 10^-9 ^(m)
*L*	Pore length	5 × 10^-6 ^(m)
*k*	Surface reaction rate constant	5 × 10^-4^, 5 × 10^-5^, 5 × 10^-7 ^(m/s)
*M*_*B*_	Molecular weight of water	18
*R*	Gas constant	8.31 (J/K. mol)
*F*	Faraday constant	9.648 × 10^4 ^(C/mol)
*D*_*F*_	Diffusivity of ferro- and ferri-cyanide	8 ×10^-10^m^2^/s



where A represents the solute (e.g., glucose or the mediator) and B the solvent (e.g., water), *ε*_*B *_the association factor of the solvent, *M*_*B *_the molecular weight of the solvent, *μ *the viscosity of solution, *V*_*A *_the molar volume of solute glucose, and *T *the absolute temperature.

## Results and Discussion

**Figure 2:**
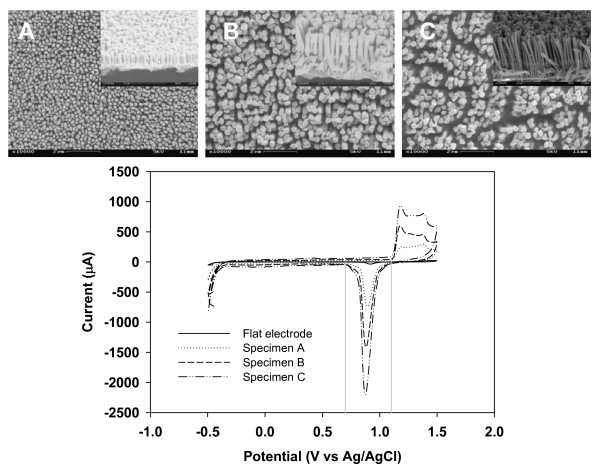
SEM images of Nano A, B and C specimens with insets showing a side-view of the specimens. Also shown here are the cyclic voltammograms obtained for three bare NAEs and a flat electrode.

Figure 2 shows three SEM images of electrodes Nano A, B and C. In all specimens, the nanopillars had a diameter of about 150 nm. From the side views (see insets in Fig. 2) of the specimens, the height of the nanopillars is estimated to be 1 μm, 2.5 μm and 6 μm for specimen A, B and C, respectively. From these images, we noted that in electrodes with taller nanopillars (e.g., Nano B and Nano C) there are slight bunching deformations in nanopillars. This kind of deformation is caused by the capillary interaction (during the wetting of the electrodes) compounded by the reduced flexure rigidity of these taller nanopillars [12]. Also shown here is the cyclic voltammograms for three NAEs and a flat electrode measured in 0.3 M sulphuric acid. In all these voltammograms, a reduction peak is seen in between 0.70 V and 1.1 V. To quantify the difference in the height of the nanopillars in these NAEs, we defined a roughness ratio as the area under the reduction peak (calculated by integrating the voltammogram from 0.70 V to 1.1 V) of a NAEs electrode divided by that of the flat electrode. The roughness ratio was found to be about 20, 38.8 and 63.4 for specimens A, B and C, respectively (see Table 2).

**Table 2:** The roughness ratio, detection sensitivity, *I*_max _and *K*_*m *_obtained from experiments

Specimen	Roughness Ratio	Sensitivity of bare electrodes to K_4_Fe(CN)_6 _(μA·mM^-1^·cm^-2^)	Sensitivity of functionalize electrodes to glucose (μA·mM^-1^·cm^-2^)	*I*_max _glucose (μA)	*K*_*m *_glucose (mM)
Flat	1.0	19.30	0.27	1.34	24.8
Nano A	20.0	41.40	0.91	5.06	29.3
Nano B	38.8	41.05	1.80	10.1	32.6
Nano C	63.4	41.70	3.13	23.0	52.0

**Figure 3:**
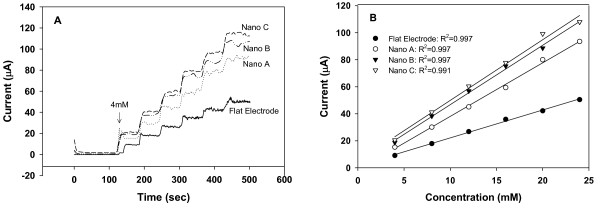
(A) Amperometric current responses obtained for the bare NAEs and flat electrode when incremental drops of K_4_Fe(CN)_6 _were added to the solution. (B) Calibration curves obtained based on a linear-regression analysis for the relationship between the steady-state current and K_4_Fe(CN)_6 _concentration.

Figure 3A shows the amperometric current response for the bare electrodes (NAEs and flat) at various K_4_Fe(CN)_6 _concentrations. In general, all the NAEs exhibited a higher current than the flat electrode at each K_4_Fe(CN)_6 _concentration. To further quantify the sensing performance of these bare electrodes, we analyzed the relationship between the current response and K_4_Fe(CN)_6 _concentration by a linear regression analysis. Figure 3B shows the variation of the steady-state amperometric current with the concentration of K_4_Fe(CN)_6 _(from 4 mM to 24 mM) along with the corresponding regression lines. By taking the slope of the regression lines and normalizing it with respect to the geometrical area of the electrodes (3.2 mm × 3.2 mm), we obtained sensitivity values for the electrodes and these values were listed in Table 2. Clearly, for all the bare electrodes, the NAEs showed sensitivity about two times higher than that of the flat electrode.

One surprising observation, however, was that the sensitivity of these bare NAEs did not increase with the increase of the roughness ratio. This implies that the benefit of the increased surface area due to nanopillars has not been fully realized. It seems that only the top part of the nanopillars has contributed to the increase of active electrode surface for electron transfer, which may explain why there is only a two-fold increase in the current responses of all the NAEs as compared with the flat electrode. We speculate that the electroactive species K_4_Fe(CN)_6 _may encounter certain difficulties in its transport to the small spaces between the bare nanopillars as the result of either a low diffusivity or a fast electron transfer rate constant. With a low diffusivity, it would be difficult for K_4_Fe(CN)_6 _to diffuse deep into the small spaces between the nanopillars, while with a fast electron transfer rate constant, most of the species K_4_Fe(CN)_6 _would get oxidized near the top ends of the nanopillars before it diffuses down the gaps. Under such a circumstance, it is conceivable that only the top regions of the nanopillars are serving their active duty in transferring electrons.

**Figure 4:**
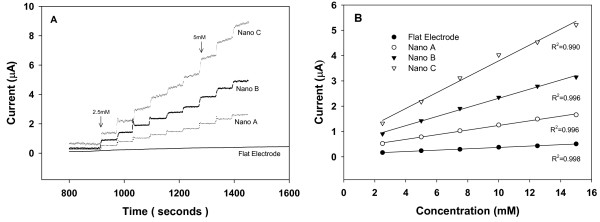
(A) Amperometric current responses obtained for the functionalized NAEs and flat electrode when incremental drops of glucose were added to the solution. (B) Calibration curve obtained based on a linear-regression analysis for the relationship between the steady-state current and glucose concentration (from 2.5 mM to 15 mM).

Figure 4A shows the amperometric currents for the functionalized NAEs and flat electrode at various glucose concentrations. Again, all the NAEs exhibited a higher current response than the flat electrode at each glucose concentration. Note that in each incremental step, the current response of Nano C is still rising indicating that it has not reached its steady state. We believe this phenomenon is due to the increased response times for electrodes with taller nanopillars. However, for a quick comparison between these nano electrodes, we took a more conservative approach to get the current readings for Nano C at the same time as for Nano B and Nano C.

Figure 4B shows the variations of the steady-state amperometric current with glucose concentration (from 2.5 mM to 15 mM) along with the corresponding linear regression lines. By taking the slope of the regression lines and normalizing it with respect to the geometric area of the electrode in each case, we obtained the sensitivity measurement for the functionalized electrodes (NAEs and flat). From the obtained sensitivity values listed in Table 2, we observed that unlike in the bare electrode cases, the sensitivity of NAEs increases as the roughness ratio increases. The highest sensitivity value (Nano C) is about 3.13 μA·mM^-1^·cm^-2 ^(about 12 times higher than that for a flat electrode) which is significantly higher than the value reported for a gold nanotube electrode (0.4 μA·mM^-1^·cm^-2^) [8]. So for the functionalized NAEs, increasing the surface roughness of the NAEs does contribute to an increase in detection sensitivity.

**Figure 5:**
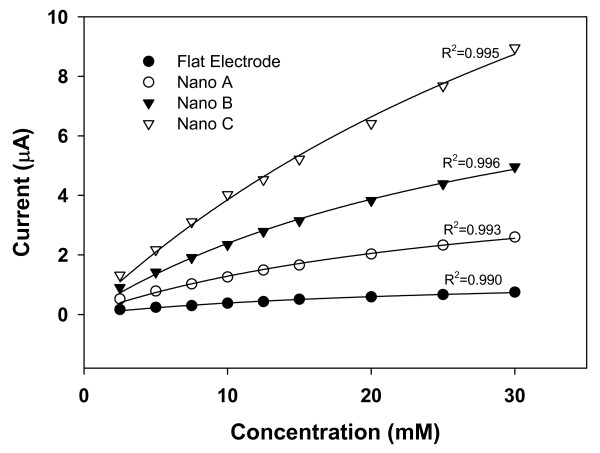
Variation of the steady-state current with glucose concentration (from 2.5 mM to 30 mM) for various functionalized electrodes along with the nonlinear-fitted curves based on the Michaelis-Menten equation.

Figure 5 shows the variations of the steady-state amperometric current with the glucose concentration over a wider concentration range (2.5 mM to 30 mM). By performing nonlinear curve fitting to the data using Eq.4, we obtained values for *K*_*m *_and *I*_max _in each case as listed in Table 2. Clearly, both *I*_max _and *K*_*m *_values are higher for the NAEs than for the flat electrode and they increase as the roughness ratio increases. Furthermore, the *K*_*m *_values for all the NAEs are larger than the reported intrinsic *K*_*m *_value of 25 mM for dissolved glucose oxidase [17]. This indicates that the activity of the enzyme immobilized on these NAEs has actually been lowered as compared with the freely dissolved enzyme, which further suggests that the increase in sensitivity in the functionalized NAEs is due to factors other than enzyme activity.

In comparing the bare with the functionalized electrodes, we found that the highest nanostructure-induced sensitivity increase for the functionalized electrodes (12 times) is higher than that for the bare electrodes (2 times). This could be due to the difference in electrochemical species involved (i.e., glucose versus K_4_Fe(CN)_6_). These two electroactive species, however, have a similar diffusivity value (8 × 10^-10^m^2^/s for K_4_Fe(CN)_6 _and 7.6 × 10^-10^m^2^/s for glucose) [15]. This fact suggests that the difference in the reaction rate constant at the bare and functionalized electrodes may play a more dominate role in affecting the current response. It is also possible that such an increase in the sensitivity of functionalized NAEs is the result of heightened retention of the mediator during glucose detection [11].

**Figure 6:**
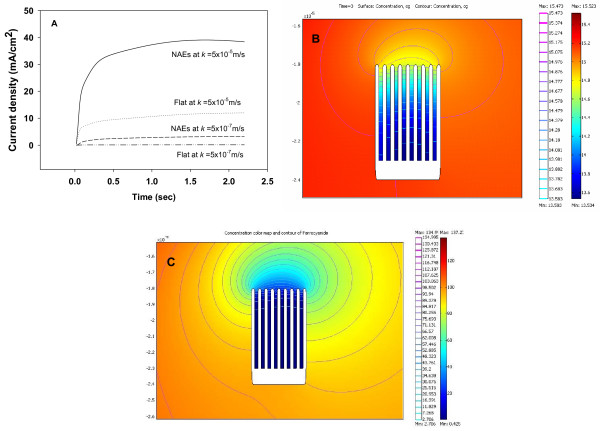
(A) Simulated current responses for a functionalized NAEs electrode and a flat electrode at a reaction rate constant of 5 × 10^-5 ^m/s and 5 × 10^-7 ^m/s. (B) Contour plot for glucose concentration near the electrode at a reaction rate constant of 5 × 10^-7 ^m/s. (C) Contour plot for K_4_Fe(CN)_6 _concentration near the electrode at a reaction rate constant of 5 × 10^-4 ^m/s.

To see the influence of the reaction rate constant on the current response of the NAEs, we now turn to the simulation results. Figure 6A shows the simulated amperometric current obtained for a functionalized NAEs and a flat electrode in response to glucose at two different reaction rate constants: 1.5 × 10^-5 ^and 1.5 × 10^-7 ^(m/s). As expected, a higher current response was found for the nanopillar electrode than for the flat electrode (see Table 3). But the nanostructure-induced increase in the current response was affected significantly by the reaction rate constant of glucose. At a rate constant of 1.5 × 10^-5 ^m/s the increase in current due to nanopillars was only 3.26 fold, whereas at a rate constant of 1.5 × 10^-7 ^m/s the increase was 22.26 fold. This fact suggests that at a lower reaction rate constant more glucose will be able to diffuse into the deep space between the nanopillars to get oxidized, thus leading to a higher current response. By contrast, K_4_Fe(CN)_6 _has a rate constant of 1.5 × 10^-4^, and at this rate constant the nanostructure-induced increase in current response is found to be only 1.28 fold (see Table 3). This is so because at such a high reaction rate constant, K_4_Fe(CN)_6 _will get oxidized quickly at the top regions of the nanopillars before it can diffuse down to the space between the nanopillars. These arguments were supported by the fact that a higher glucose concentration was found at the bottom of the spaces between nanopillars in the case with a lower reaction rate constant: a concentration of 0.497 mol/m^3 ^and 13.583 mol/m^3 ^was found at the bottom of the spaces between nanopillars when the rate constant is 1.5 × 10^-5 ^m/s and 1.5 × 10^-7 ^m/s, respectively. Figure 6B shows a contour plot for glucose concentration at a rate constant of 1.5 × 10^-7^m/s, where it is seen that a significant amount of glucose reached to the bottom of the spaces between nanopillars. In the case of K_4_Fe(CN)_6 _its concentration is found to be zero at the bottom of the spaces between nanopillars (see Figure 6C).

**Table 3:** Steady-state amperometric current density obtained at different rate constants from computer simulation

Reaction rate constant (m/s)	Current Density (mA·cm^-2^)		
	
	Nano	Flat	Nano/Flat
5 × 10^-4 ^for K_4_Fe(CN)_6_	279.51	219.18	1.28
5 × 10^-5 ^for glucose	39.1	12.0	3.26
5 × 10^-7 ^for glucose	3.25	0.146	22.26

The results obtained from simulation are consistent with the prediction for the catalytic reactions in porous media based on the effectiveness factor (*η*) and thiele modulus (*φ*) concepts. The value of *η *can be determined by using the following formula [18]:

{η≈1/φl,ifφs2<<1andφl2>>1η≈1,ifφs2<<1andφl2<<1
 MathType@MTEF@5@5@+=feaafiart1ev1aaatCvAUfKttLearuWrP9MDH5MBPbIqV92AaeXatLxBI9gBaebbnrfifHhDYfgasaacH8akY=wiFfYdH8Gipec8Eeeu0xXdbba9frFj0=OqFfea0dXdd9vqai=hGuQ8kuc9pgc9s8qqaq=dirpe0xb9q8qiLsFr0=vr0=vr0dc8meaabaqaciaacaGaaeqabaqabeGadaaakeaadaGabaqaauaabeqacyaaaaqaaGGaciab=D7aOjabgIKi7kabigdaXiabc+caViab=z8aMnaaBaaaleaacqWGSbaBaeqaaOGaeiilaWcabaaabaGaemyAaKMaemOzaygabaGae8NXdy2aa0baaSqaaiabdohaZbqaaiabikdaYaaakiabgYda8iabgYda8iabigdaXaqaaiabdggaHjabd6gaUjabdsgaKbqaaiab=z8aMnaaDaaaleaacqWGSbaBaeaacqaIYaGmaaGccqGH+aGpcqGH+aGpcqaIXaqmaeaacqWF3oaAcqGHijYUcqaIXaqmcqGGSaalaeaaaeaacqWGPbqAcqWGMbGzaeaacqWFgpGzdaqhaaWcbaGaem4CamhabaGaeGOmaidaaOGaeyipaWJaeyipaWJaeGymaedabaGaemyyaeMaemOBa4MaemizaqgabaGae8NXdy2aa0baaSqaaiabdYgaSbqaaiabikdaYaaakiabgYda8iabgYda8iabigdaXaaaaiaawUhaaaaa@66CD@

Here φs2
 MathType@MTEF@5@5@+=feaafiart1ev1aaatCvAUfKttLearuWrP9MDH5MBPbIqV92AaeXatLxBI9gBaebbnrfifHhDYfgasaacH8akY=wiFfYdH8Gipec8Eeeu0xXdbba9frFj0=OqFfea0dXdd9vqai=hGuQ8kuc9pgc9s8qqaq=dirpe0xb9q8qiLsFr0=vr0=vr0dc8meaabaqaciaacaGaaeqabaqabeGadaaakeaaiiGacqWFgpGzdaqhaaWcbaGaem4CamhabaGaeGOmaidaaaaa@30FA@ and φl2
 MathType@MTEF@5@5@+=feaafiart1ev1aaatCvAUfKttLearuWrP9MDH5MBPbIqV92AaeXatLxBI9gBaebbnrfifHhDYfgasaacH8akY=wiFfYdH8Gipec8Eeeu0xXdbba9frFj0=OqFfea0dXdd9vqai=hGuQ8kuc9pgc9s8qqaq=dirpe0xb9q8qiLsFr0=vr0=vr0dc8meaabaqaciaacaGaaeqabaqabeGadaaakeaaiiGacqWFgpGzdaqhaaWcbaGaemiBaWgabaGaeGOmaidaaaaa@30EC@ are the thiele moduli calculated based on the transverse and longitudinal diffusion times, and they are defined by φs2
 MathType@MTEF@5@5@+=feaafiart1ev1aaatCvAUfKttLearuWrP9MDH5MBPbIqV92AaeXatLxBI9gBaebbnrfifHhDYfgasaacH8akY=wiFfYdH8Gipec8Eeeu0xXdbba9frFj0=OqFfea0dXdd9vqai=hGuQ8kuc9pgc9s8qqaq=dirpe0xb9q8qiLsFr0=vr0=vr0dc8meaabaqaciaacaGaaeqabaqabeGadaaakeaaiiGacqWFgpGzdaqhaaWcbaGaem4CamhabaGaeGOmaidaaaaa@30FA@ = 2*kr*_*p*_/*D*_*G*_, φl2
 MathType@MTEF@5@5@+=feaafiart1ev1aaatCvAUfKttLearuWrP9MDH5MBPbIqV92AaeXatLxBI9gBaebbnrfifHhDYfgasaacH8akY=wiFfYdH8Gipec8Eeeu0xXdbba9frFj0=OqFfea0dXdd9vqai=hGuQ8kuc9pgc9s8qqaq=dirpe0xb9q8qiLsFr0=vr0=vr0dc8meaabaqaciaacaGaaeqabaqabeGadaaakeaaiiGacqWFgpGzdaqhaaWcbaGaemiBaWgabaGaeGOmaidaaaaa@30EC@ = 2*kL*^2^/*r*_*p*_*D*_*G*_, where *r*_*p *_is the pore radius, *L *is the pore length, *D*_*G *_is the glucose diffusivity and *k *is the surface rate constant. For a surface rate constant of 5 × 10^-4 ^m/s, we calculated φs2
 MathType@MTEF@5@5@+=feaafiart1ev1aaatCvAUfKttLearuWrP9MDH5MBPbIqV92AaeXatLxBI9gBaebbnrfifHhDYfgasaacH8akY=wiFfYdH8Gipec8Eeeu0xXdbba9frFj0=OqFfea0dXdd9vqai=hGuQ8kuc9pgc9s8qqaq=dirpe0xb9q8qiLsFr0=vr0=vr0dc8meaabaqaciaacaGaaeqabaqabeGadaaakeaaiiGacqWFgpGzdaqhaaWcbaGaem4CamhabaGaeGOmaidaaaaa@30FA@ = 0.131 and φl2
 MathType@MTEF@5@5@+=feaafiart1ev1aaatCvAUfKttLearuWrP9MDH5MBPbIqV92AaeXatLxBI9gBaebbnrfifHhDYfgasaacH8akY=wiFfYdH8Gipec8Eeeu0xXdbba9frFj0=OqFfea0dXdd9vqai=hGuQ8kuc9pgc9s8qqaq=dirpe0xb9q8qiLsFr0=vr0=vr0dc8meaabaqaciaacaGaaeqabaqabeGadaaakeaaiiGacqWFgpGzdaqhaaWcbaGaemiBaWgabaGaeGOmaidaaaaa@30EC@ = 328.8. Under this condition, *η *is found to be *η *≈ 1/*ϕ *= 0.055. This low effectiveness factor will hinder the transport of the target species to the spaces between the nanopillars. For a lower surface reaction rate constant of 5 × 10^-7 ^m/s, we calculated φs2
 MathType@MTEF@5@5@+=feaafiart1ev1aaatCvAUfKttLearuWrP9MDH5MBPbIqV92AaeXatLxBI9gBaebbnrfifHhDYfgasaacH8akY=wiFfYdH8Gipec8Eeeu0xXdbba9frFj0=OqFfea0dXdd9vqai=hGuQ8kuc9pgc9s8qqaq=dirpe0xb9q8qiLsFr0=vr0=vr0dc8meaabaqaciaacaGaaeqabaqabeGadaaakeaaiiGacqWFgpGzdaqhaaWcbaGaem4CamhabaGaeGOmaidaaaaa@30FA@ = 131.5 × 10^-6 ^and φl2
 MathType@MTEF@5@5@+=feaafiart1ev1aaatCvAUfKttLearuWrP9MDH5MBPbIqV92AaeXatLxBI9gBaebbnrfifHhDYfgasaacH8akY=wiFfYdH8Gipec8Eeeu0xXdbba9frFj0=OqFfea0dXdd9vqai=hGuQ8kuc9pgc9s8qqaq=dirpe0xb9q8qiLsFr0=vr0=vr0dc8meaabaqaciaacaGaaeqabaqabeGadaaakeaaiiGacqWFgpGzdaqhaaWcbaGaemiBaWgabaGaeGOmaidaaaaa@30EC@ = 0.328, and under this condition *η *will be close to unity (*η *≈ 1). This high effectiveness factor will surely enable more efficient transport of glucose to the functionalized surfaces in between the nanopillars.

The above results clearly indicate that the enhanced current response in glucose sensing with functionalized NAEs can be attributed to the effective mass transport facilitated by the relatively lower reaction rate constant of glucose. This fact suggests that to reap the true benefit of using nanostructured electrodes for enhancing the performance of biosensors, it is necessary to optimize the geometry of the nanopillars (their diameter, spacing and height) in order to accommodate the specific analyte species in terms of its reaction kinetics and mass transport.

## Conclusion

The role of reaction kinetics and mass transport in biosensing using electrodes integrated with nanopillars of different heights was investigated. In an electrochemical based detection, the increased active surface area due to the addition of nanopillars may lead to enhanced sensing performances only when the reaction rate constant of the target species is low. At a higher reaction rate constant, only the top part of the nanopillar modified electrodes will serve the purpose for transferring electrons. To reap the benefit of using nanostructured electrodes for improving the sensing performances, it is necessary to optimize the geometry of the nanopillars to accommodate the specific analyte species in terms of its reaction kinetics and mass transport.

## Competing interests

The author(s) declare that they have no competing interests.

## Authors' contributions

VA, XY, EK and YLR contributed to the design of the study, the acquisition and analysis of data, and the writing of the manuscript. GZ contributed to the design and coordination of the study and participated in the writing of the manuscript. All authors read and approved the final manuscript.
